# Effects of GLP-1 agonists and SGLT2 inhibitors during pregnancy and lactation on offspring outcomes: a systematic review of the evidence

**DOI:** 10.3389/fendo.2023.1215356

**Published:** 2023-10-10

**Authors:** Dion R. P. Muller, Dirk J. Stenvers, Arjan Malekzadeh, Frederik Holleman, Rebecca C. Painter, Sarah E. Siegelaar

**Affiliations:** ^1^ Department of Endocrinology and Metabolism, Amsterdam University Medical Centers (UMC) Location University of Amsterdam, Amsterdam, Netherlands; ^2^ Amsterdam Gastroenterology Endocrinology and Metabolism, Amsterdam, Netherlands; ^3^ Medical Library, Amsterdam University Medical Centers (UMC) Location University of Amsterdam, Amsterdam, Netherlands; ^4^ Department of Internal Medicine, Amsterdam University Medical Centers (UMC) Location University of Amsterdam, Amsterdam, Netherlands; ^5^ Department of Gynaecology and Obstetrics, Amsterdam University Medical Centers (UMC) Location Vrije Universiteit Amsterdam, Amsterdam, Netherlands; ^6^ Amsterdam Reproduction and Development, Amsterdam, Netherlands

**Keywords:** diabetes, pregnancy, systematic review, GLP-1 agonist, SGLT2 inhibitor, lactation

## Abstract

**Aims/hypothesis:**

Glucagon-like peptide 1 (GLP-1) agonists and sodium-glucose co-transporter-2 (SGLT2) inhibitors are novel drugs which have recently seen rapid uptake in the treatment of type 2 diabetes and obesity. The paucity of data regarding their safety during pregnancy and lactation causes a dilemma for the physician. The aim of the present study was to systematically review all available data on the offspring effects of GLP-1 agonists and SGLT2 inhibitors during pregnancy and lactation.

**Methods:**

We systematically searched PubMed, clinicaltrials.gov, FDA and EMA product information on GLP-1 agonists and SGLT2 inhibitors in pregnancy and lactation from inception up to 19 April 2022 without language restrictions. We approached both the Netherlands Pharmacovigilance Centre Lareb on January 17^th^ 2023 and the Teratology Information Service (TIS) of Switzerland on February 6^th^ 2023. Eligible studies investigating the safety (including congenital anomalies, fetal growth, perinatal demise) in animals or humans, or reporting the degree of transfer of these drugs to the fetus, breast milk or breastfed neonate. Two reviewers independently assessed and selected studies for inclusion and subsequently resolved discrepancies by discussion.

**Results:**

We included 39 records (n=9 theoretical; based on drug properties, n=7 human; n=23 animal, including 76 human offspring, and an unknown number of animal offspring as these numbers could not be retrieved from the FDA and EMA product information). In animal studies, GLP1-agonists were associated with reduced fetal weight and/or growth, delayed ossification and skeletal variants, usually associated with a reduction in maternal weight gain and decreased food consumption. Exendin-4 (GLP1-agonist) was not transported across the maternal-fetal placental interface. In human studies, exenatide (GLP1-agonist) showed a fetal-to-maternal peptide concentration ratio of ≤ 0.017 in ex vivo human placental perfusion in a single placenta. Liraglutide (GLP1-agonist) showed no significant maternal to fetal transfer at least 3.5 hours after maternal exposure in a human study with one subject. In animal studies, GLP-1 agonists were excreted in breast milk; human data on excretion were not available. In animal studies, SGLT2 inhibitors were generally safe during the first trimester but exposure during postnatal day 21 to 90 in juvenile rats, a period coinciding with the late second and third trimester of human renal development, caused dilatation of the renal pelvis and tubules. Human data consisted of a pharmaceutical database of inadvertent pregnancies during SGLT2 inhibitor use, which found an increase in miscarriages and congenital malformations. In animal studies SGLT2 inhibitors were excreted in breast milk and affected neonatal growth, but human data are not available.

**Conclusion/interpretation:**

We found evidence for adverse offspring effects of GLP-1 agonists and SGLT2 inhibitors also in human studies. Our findings broadly support the advice to discontinue GLP-1 agonists and SGLT2 inhibitors during pregnancy and lactation, and also support the ongoing registration of pregnancy outcomes in pharmacological databases since the amount of available data is scarce and mostly limited to animal studies.

**Registration:**

https://www.crd.york.ac.uk/prospero/display_record.php?RecordID=219877

## Research in context

**What is already known about this subject?** To date there have been no systematic reviews on the safety of GLP-1 agonists and/or SGLT2 inhibitors during pregnancy or lactation.

**What is the key question?** What is the current scientific data on the safety of GLP-1 agonists and SGLT2 inhibitors during pregnancy and/or lactation?

**What are the new findings?** GLP-1 agonists affect fetal weight, growth and skeletal ossification in animal studies, although liraglutide and exenatide do not cross the placenta.

In animals, SGLT2 inhibitors cause dilatation of the renal pelvis and tubules of the kidney when used during the second and third trimester and additionally a relatively large number of unfavorable human pregnancy outcomes are found in humans.

In animal studies GLP-1 agonists and SGLT2 inhibitors are excreted in breast milk and affect growth, human data is not available.

**How might this impact on clinical practice in the foreseeable future?** At present, the available evidence suggests that both GLP-1 agonist and SGLT2 inhibitors produce unfavourable outcomes, and should be avoided in pregnancy and during lactation.

## Introduction

The presence of type 2 diabetes or maternal obesity during pregnancy increases the risk of adverse pregnancy outcomes and birth defects (1–6). The risk of these adverse outcomes can be reduced by optimal glycemic control and healthy weight (7). In pregnancy, patients with type 2 diabetes are mainly treated with insulin or metformin (7).

In a non-pregnant patient with type 2 diabetes, a GLP-1 agonist or SGLT2 inhibitor can be prescribed if adequate glycemic control is not achieved with metformin, sulphonylureas or (basal) insulin, or if weight reduction, hypoglycemia prevention or reduction of cardiovascular risk is the treatment goal (8–10). GLP-1 agonists give an average reduction of HbA1c of 11 mmol/mol in comparison with placebo (11) and SGLT2 inhibitors give an average HbA1c reduction of 7-9 mmol/mol (12). Both classes of medications cause few hypoglycemic episodes.

Despite the fact that these novel glucose lowering medications have seen wide uptake in clinical practice, information regarding the safety of GLP-1 agonists and SGLT2 inhibitors during pregnancy and lactation is exceedingly scarce. As these drugs are being used by an increasing group of fertile women, it is expected that a few of them will get pregnant and data on the safety of continuation would prove valuable. Therefore, the aim of the present study is to combine all available data on the safety of these medication classes during pregnancy and lactation.

## Methods

### Protocol

The study protocol for the present systematic review was first published 23 January 2021 in the PROSPERO database, https://www.crd.york.ac.uk/prospero/display_record.php?RecordID=219877.

### Protocol deviations

The following deviations from the initial protocol were necessary and were amended in a protocol update: the search strategy was updated to include Embase and Cochrane. Furthermore we approached both the Netherlands Pharmacovigilance Centre Lareb on January 17^th^ 2023 and the Teratology Information Service (TIS) of Switzerland on February 6^th^ 2023.

### Search strategy

We created a search syntax for 3 databases; Medline, Embase and Cochrane, to search for studies during pregnancy and/or lactation with maternal exposure to a GLP-1 agonist or an SGLT2 inhibitor (**Supplement 4–6**). We included both animal and human studies including case reports. Data was retrieved for studies from inception up to 19 April 2022.

The international trial register clinicaltrials.gov was searched for published and unpublished studies by searching each of the individual drugs combined with the terms pregnancy, diabetes mellitus, reproduction and lactation. The product information of the Food and Drug Administration (FDA, accessdata.fda.gov) and European Medicine Agency (EMA, ema.europa.eu) on all GLP-1 agonists and SGLT2 inhibitors were searched for additional data. All GLP-1 agonists, SGLT2 inhibitors and their availability are described in **Supplement 1**.

Furthermore, we approached both Netherlands Pharmacovigilance Centre Lareb on 17 January 2023 and the Teratology Information Service (TIS) of Switzerland on 6 February 2023 for any pregnancy outcomes of women exposed to either GLP-1 agonists or SGLT2 inhibitors during pregnancy available to them.

### Study selection

All types of studies that met one of the following criteria were included: (1) papers describing the fetal/neonatal pregnancy outcomes on the level of congenital anomalies, fetal growth and perinatal demise of maternal exposure to a GLP-1 agonist or an SGLT2 inhibitor during pregnancy or lactation; (2) articles regarding transplacental transfer or drug concentrations *in utero* or postpartum in fetus or neonate, breast milk concentration, or drug concentrations in nursing infants. Two authors (DM and SS) independently reviewed and selected studies for inclusion and subsequently resolved discrepancies by discussion.

### Data extraction and quality assessment

A single reviewer (DM) undertook data extraction and quality assessment. Data were extracted as follows: (1) species, number exposed to drug of interest, number of controls, timing and duration of exposure, dose (drug/kg/time-interval and equivalents to maximum recommended human dosage or clinical dosage), fetal effects of maternal GLP-1 agonist or SGLT2 inhibitor exposure, (2) species, number exposed to drug of interest, number of controls, timing and duration of exposure, dose (drug/kg/time-interval and equivalents to maximum recommended human dosage or clinical dosage), the transfer of GLP-1 agonist or SGLT2 inhibitor in maternal milk and the effect of exposure during lactation on the neonate. These data were subsequently translated to tables describing the fetal effects of maternal exposure observed for each drug. For animal studies quality assessment was performed using SYRCLE’s risk of bias tool (13).

## Results

**Figure 1:**
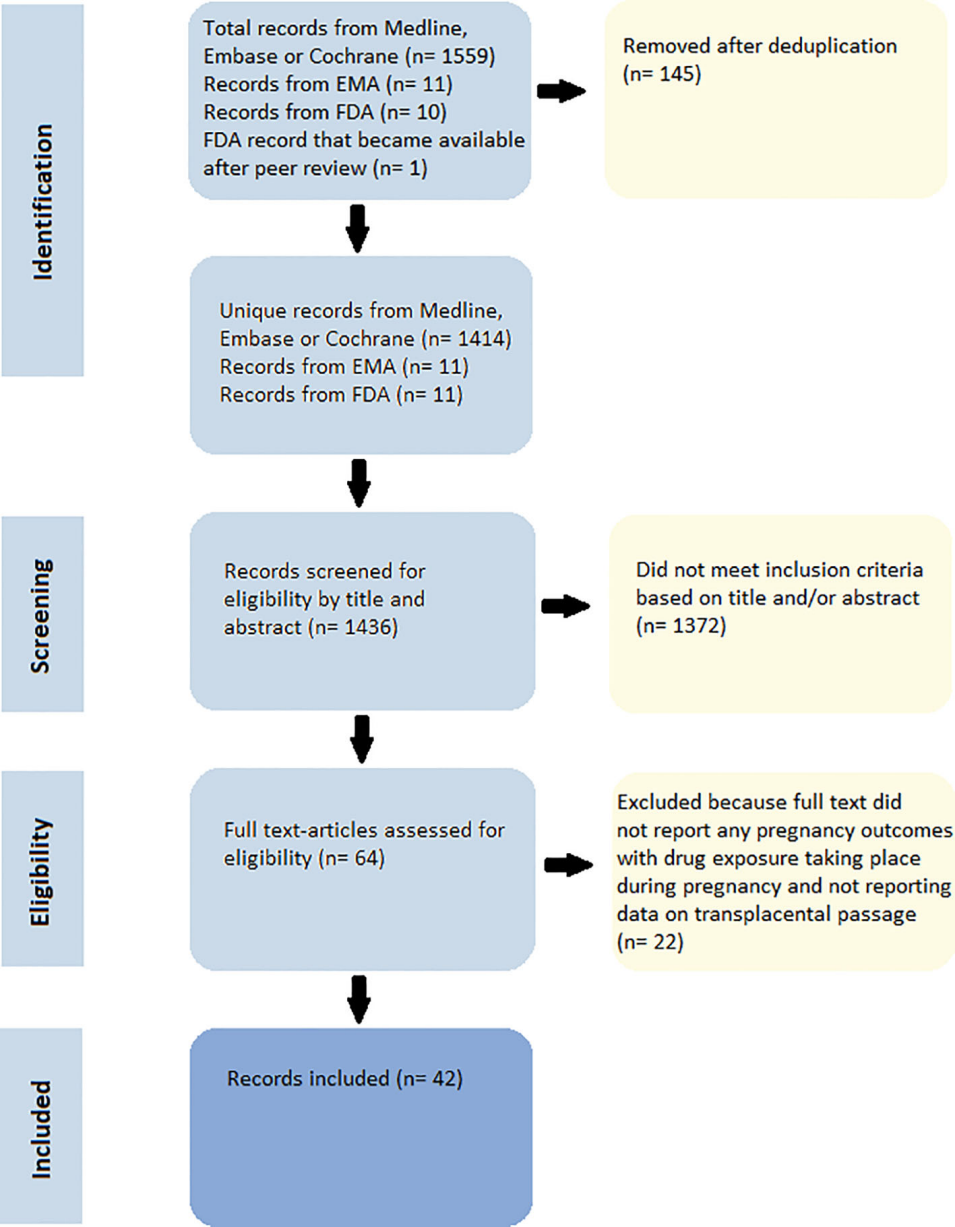
PRISMA flowchart.

Out of a total of 1436 identified unique records (**Figure 1**), we excluded 1372 records for not meeting the inclusion criteria based on title or abstract or upon further evaluation of the full text. We included 26 records regarding the safety of GLP-1 agonists during pregnancy and/or lactation (**Supplement 2**, **3**), and 16 records regarding the safety of SGLT2 inhibitors during pregnancy and/or lactation (**Supplement 2**, **3**). All the product information data for the GLP-1 agonists and SGLT2 inhibitors from both the EMA and the FDA databases was extracted and included. No double records were found. All animal studies, including the EMA and FDA records, had a high risk of bias (**Supplement 7**). No data was received through our contact with both the Lareb and TIS of Switzerland as the pregnancy database of the Lareb currently does not contain any women that have used either GLP-1 agonists or SGLT2 inhibitors and on 16 March 2023 the TIS is in the process of receiving and analyzing data from multiple European Network of Teratology Information Services (ENTIS) centers on women exposed to GLP-1 agonists during pregnancy and did not have any preliminary/extracted data available to share.

### GLP-1 agonists during pregnancy

#### Animal

Exendin-4, of which exenatide is the synthetic form, was the only GLP-1 agonist for which any data regarding placental passage was available. The included studies on exendin-4 exposure were all performed in mouse models (14, 15). When healthy pregnant mice were injected once with fluorescence labeled exendin-4 on gestation day 16. One day later, after euthanasia, the exendin-4 was not detected in any of the fetal tissues (fetus, decidua, placenta, fetal membranes) or maternal uterus. However, in mice with systemic inflammation, exendin-4 was strongly detected in the uterus and traces were also found in the decidua and fetal membranes (14). The other mouse study reported no difference in offspring weight at birth or maternal weight gain during pregnancy but did observe an increase in pre-weaning fetal growth after maternal exendin-4 (15).

In rat, mouse and rabbit models without systemic inflammation, the other GLP1-receptor agonists (except exendin-4), dose dependently caused reduced fetal weight and/or growth, retardation of ossification, irregular ossification, and/or skeletal variants such as wavy ribs (**Table 1**). These effects were mostly accompanied by decreased maternal food consumption and a reduction in maternal weight. Reduced embryonic survival was associated with maternal weight loss. In rats, more severe effects were seen after maternal exposure to semaglutide in the form of visceral abnormalities and major skeletal malformations (**Table 1**).

**Table 1:** Animal data on fetal effects of maternal GLP-1 agonist exposure.

Drug	Reduced embryonic survival	Decreased fetal weight/growth	Increased pre-weaning fetal growth	Delayed ossification and skeletal variations*	Visceral* congenital abnormalities	Major skeletal congenital malformations
Liraglutide	+^C^	+^A,B,C^		+^A,B^	+^B^	–
Exendin-4	–	–	+^D^	–	–	
Exenatide	–	+^A,B^		+^A,B^	–	–
Albiglutide	+^A,B^	+^A,B^		+^A,B^	–	–
Dulaglutide	+^A,B^	+^A,B^		+^A,B^	–	–
Lixisenatide	–	+^A^		+^A,B^	–	–
Semaglutide	+^A,B^	+^A^		+^A^	+^A^	+^A^

+ = observed, - = not observed, A = EMA product information (16–21), B = FDA product information (22–27), C = Younes et al. (28), D = Graham et al. (15) One study, Diz-Chaves et al. found no adverse fetal effects (29). *(Skeletal) variations: structural change that does not impact viability, development, or function (e.g.wavy ribs) that can be found in the normal population under investigation and can be reversible, **Visceral: Having to do with the viscera; which are the soft internal organs of the body, including the lungs, heart, and the organs of the digestive, excretory, reproductive, and circulatory systems.

#### Human

An earlier review on pharmacotherapy for gestational diabetes reported no human data on the safety of GLP-1 agonists during pregnancy and concluded that these drugs cannot be recommended in pregnancy (30). We identified a case report of a 37-year-old woman with type 2 diabetes on liraglutide in the 13^th^ week of gestation (31). After pregnancy confirmation, liraglutide was discontinued and insulin therapy was started. She delivered a healthy child after an uneventful gestation. Another case report of a 28-year-old woman with type 2 diabetes treated with liraglutide and metformin for the full duration of her pregnancy until elective caesarean section at 39 gestational weeks was found (32). After delivery, 3.5 hours after the latest dose of liraglutide, the concentration of liraglutide was measured in maternal and umbilical vein serum with an active GLP-1 ELISA. After removal of endogenous GLP-1 by thermal degradation the levels of liraglutide in maternal serum were found to be 8.4 pmol/l. In the umbilical vein blood GLP-1 levels were found to be 0.7 pmol/l which is below the limit of sensitivity of the assay declared by the manufacturer of 2 pmol/l and was similar to the GLP-1 levels found in the umbilical vein blood of the control (a healthy pregnant woman not exposed to liraglutide). These findings imply that, in term pregnancy, there was no significant maternal transfer of liraglutide to the fetus at least 3.5 hours after administration. A final case report of a woman with type 2 diabetes on exenatide, rosiglitazone, metformin, and glargine insulin in the 14^th^ week of gestation was identified (33). After confirmation of the pregnancy her glucose lowering medication was converted to insulin glargine and lispro. In this case, after an uneventful pregnancy, the patient delivered an anatomically normal fetus despite exposure to exenatide during the period of organogenesis. The only other human study was an *ex vivo* equilibrium perfusion experiment of human placentas with exenatide that measured a fetal-to-maternal peptide concentration ratio of ≤ 0.017 meaning negligible placental passage (34).

### SGLT2 inhibitors during pregnancy

#### Animal

None of the SGLT2 inhibitors showed developmental toxicity when a dose not resulting in maternal toxicity was administered during the interval coinciding with the first trimester period of organogenesis except for canagliflozin, where ossification delays of the metatarsal bones were seen (**Table 2**).

**Table 2:** Animal data on fetal effects of maternal SGLT2 inhibitor exposure.

Drug	Developmental toxicity^C^	Dilatation of renal pelvis and tubule^D^	Reversibility of renal changes
Canagliflozin	Ossification delays of metatarsal bones ^A^.	+^A,B^	Not fully reversible ^A,B^
Empagliflozin	–	+^A,B^	Reversible^A,B^
Dapagliflozin	–	+^A,B^	Not fully reversible ^A,B^
Ertugliflozin	–	+^A,B^	Not fully reversible ^A,B^
Sotagliflozin	–	+^A,B^	Reversible^A^
Ipragliflozin	No EMA/FDA data		
Luseogliflozin			
Remogliflozin			
Sergliflozin			
Tofogliflozin			

+ = observed, - = not observed, A = EMA product information (35–39), B = FDA product information (40–44), C = when given during period of organogenesis, at doses below maternal toxicity, D = when given during period corresponding to late second and third trimesters in humans.

#### Human

Human data was identified in the form of a case report on SGLT2 inhibitor use during pregnancy of a 31-year-old woman with type 2 diabetes on empagliflozin, metformin and insulin degludec who was found to be pregnant at the 5^th^ week of gestation (45). After pregnancy confirmation, empagliflozin was discontinued immediately and metformin was discontinued after the first trimester. She gave birth to a healthy child without congenital malformations. Furthermore, a study describing the data in the pharmaceutical company safety databases on pregnant women exposed to glucose lowering drugs including SGLT2 inhibitors was found (46). For Canagliflozin 29 pregnancies were reported of which 14 listed the corresponding pregnancy outcomes:1 congenital anomaly, 2 abortions, 1 fetal demise, 8 normal live births, 2 ongoing pregnancies. For Empagliflozin 21 pregnancies were reported of which 8 listed the corresponding pregnancy outcomes: 2 spontaneous abortions, 1 elective termination, 1 ectopic pregnancy, 3 healthy infants without congenital anomaly, 1 premature infant. For Dapagliflozin 21 pregnancies were reported of which 8 listed the corresponding pregnancy outcomes: 1 congenital hydrocephalus, 2 encephalocele, 1 non-healthy infant with talipes, renal aplasia and oesophageal atresia, 2 induced abortions, 1 healthy premature infant, 1 healthy infant.

### GLP-1 agonists during lactation

#### Animal

The concentration of exenatide in the milk of mice was ≤2.5% of the maternal plasma concentration. These ratios in exposed rats were 8.3% - 33% for semaglutide, 9.4% for lixisenatide and 50% for liraglutide. Concentrations in milk were not determined for dulaglutide and albiglutide (**Supplement 3**). All GLP-1 agonists caused reduced fetal growth and decreased weight when used during late gestation and lactation. A reduction in neonatal growth was also seen when liraglutide was used during lactation without having been used during pregnancy (**Supplement 3**).

#### Human

No human studies regarding the safety of GLP-1 agonists during lactation were found. In theory, the transfer to human milk of GLP-1 agonists may be low, as they are large peptide molecules (47–51) (**Supplement 3**). However low, a certain degree of excretion in human milk is expected as animal data is a reliable predictor of the presence of a drug and/or its active metabolite(s) in human milk (52). GLP-1 agonists that are ingested by the infant might be digested in the infants’ gastrointestinal tract (**Supplement 3**) but the animal data suggest a systemic effect.

### SGLT2-inhibitors during lactation

#### Animal

All SGLT2 inhibitors are excreted in milk in animal studies (**Supplement 3**). Exposure to SGLT2 inhibitors from postnatal day 21 to 90, the period functionally coinciding with the late second and third trimesters of human pregnancy regarding kidney development, caused dilatation of the renal pelvis and tubules (**Table 2**). These changes persisted or were only partially reversible except for the changes caused by empagliflozin and sotagliflozin which showed full reversibility upon discontinuation.

#### Human

No studies regarding the use of SGLT2 inhibitors during lactation in humans were found. On theoretical grounds, excretion in human milk should be low since these molecules are uncharged and highly protein bound in plasma, their molecular weight is fairly low however ± 400 Da (12, 53–55) (**Supplement 3**). However, SGLT-2 inhibitors are also expected to be present in human milk for they are excreted in milk in animal studies (52).

## Discussion

Human data on the fetal effects of maternal GLP-1 agonists and SGLT2 inhibitors are scarce. The available data concerns low numbers of exposed pregnancies and lack a clear description of maternal and treatment characteristics with potential effect on neonatal outcome such as maternal BMI, drug dose and trimester of exposure. The lack of data regarding exposure specifically during the first trimester is unfortunate.

The reviewed data show that maternal exposure to GLP-1 agonists causes reduced embryonic-fetal survival, increases in major congenital anomalies, reduced fetal weight/growth, retardation of ossification, irregular ossification and skeletal variants in mice, rats and rabbits. In contrast with other GLP-1 agonists, maternal exposure to exendin-4 resulted in an increase in pre-weaning fetal growth in one study (15). If this apparent difference between GLP-1 agonists is mediated by characteristics of exendin-4, a species-specific effect, or due to the dosage remains to be elucidated, as the included studies have only been performed in mice and in a small range of concentrations (14, 15). SGLT2 inhibitors cause adverse effects in the form of dilatation of the renal pelvis and tubules in the fetus of rats and rabbits, when taken during the stage coinciding with the second and third trimester of human renal development. The degree of reversibility depends on the specific drug. No human data on the excretion of GLP-1 agonists or SGLT2 antagonists in milk are available, but in rat and mouse models excretion in milk was observed with concomitant effects on neonatal growth of GLP-1 agonists. Our review is supportive of the current recommendation against the use of either of these medications during pregnancy or lactation as either metformin or a variety of insulin analogs are safe during pregnancy (56, 57) and lactation (58, 59).

The fetal effects of maternal exposure to GLP-1 agonists were marked. Maternal weight loss during GLP-1 agonists treatment during gestation was also marked. Since albumin does not cross the hemochorial placenta, as shown in the rat (60), guinea pig (61) and *in vitro* in a human placenta (62), it seems unlikely that the albumin fusion protein albiglutide should cross the placenta. Thus, the fetal effects of maternal exposure to albiglutide are more likely due to the maternal side effects of reduced maternal intake and weight loss. This is probably also the case for liraglitude where GLP-1 measurements on both the maternal and fetal compartment after maternal exposure to liraglutide did not show significant transfer to the fetus (32). Similarly, the fetal effects of exenatide, which has a negligible fetal-to-maternal peptide concentration ratio during *ex vivo* human placental perfusion, are probably due to maternal side effects (34). The low placental transfer could be caused by the release of Dipeptidyl peptidase IV positive syncytiotrophoblast-derived extracellular vesicles released by the human placenta which degrade GLP-1 in uterine blood (63), as well as to the high molecular weight of GLP-1 agonists (4 – 73 kDa). Compounds with a molecular weight <500 Da readily diffuse across the placenta (64). SGLT-2 inhibitors have a fairly low molecular weight of ± 400 Da. On the other hand SGLT-2 inhibitors are highly protein bound which may in turn inhibit their placental passage. For SGLT-2 inhibitors no studies regarding placental transfer were found.

In line, exendin-4 is undetectable in fetal tissues or the maternal uterus in healthy pregnant mice. However, exendin-4 is detectable in the uterus and in trace amounts on the decidua and fetal membranes when using an animal model of systemic maternal inflammation (65). Low grade inflammation common to maternal obesity may induce effects in humans more similar to the inflammation animal models, leading to a higher rate of maternal -fetal transfer and direct effects in the fetus. Our review found insufficient evidence to investigate this notion.

Earlier animal studies with malnourished mothers reported that delayed ossification is associated with decreased maternal body weight, and maternal malnutrition or reduced intake are common pathways to decreased fetal weight/growth and ossification (66–69), but malnutrition models did not report the increase in major congenital anomalies reported after gestational exposure to GLP-1 agonists. The product information available through the EMA states that the visceral abnormalities and major skeletal malformations seen after maternal exposure to semaglutide are probably due to embryotoxicity caused by a GLP-1 receptor-mediated impairment of the nutrient supply across the rat yolk sac, which has unclear translational significance for humans due to a difference in yolk sac anatomy. Whether depletion of specific key nutrients, including vitamins (eg vitamin D or vitamin K) could result in the specific bone and visceral effects remains unclear from the data we collected in this review.

In animal studies, SGLT2 inhibitors do not cause developmental toxicity during organogenesis in the first trimester. The only exception is canagliflozin, which causes delayed ossification of the metatarsal bones that may be attributed to effects on calcium homeostasis observed in earlier studies in healthy human adults (70). The ability to cross the rat placenta has been described as low and very limited for sotagliflozin and empagliflozin respectively. The most likely cause of the renal changes seen after maternal exposure is the inability of the developing fetal kidney to handle the increased urinary volume caused by the SGLT2 inhibitor (71). In the pharmaceutical company databases miscarriages and congenital malformations were described. Even though excretion in human milk is probably low, renal effects on the offspring cannot be excluded when using SGLT2 inhibitors during lactation, as full functional maturation of the kidney may take 2 years in humans (72).

Poor glycemic control in the periconception period and in pregnancy has significant maternal and perinatal risks (1–6). GLP-1 agonists and SGLT2 inhibitors can be useful in optimizing glycemic control beyond conventional therapy (8–10). Our findings advise against the use of such medication in pregnancy and lactation, but as long as a safe wash out period is observed, they might be carefully used in pregnancy preparation for people with type 2 diabetes.

As for addressing key questions remaining in regards to this topic we first suggest future studies to more thoroughly report maternal and treatment characteristics including trimester of exposure. We suggest to update earlier studies by publishing follow-up data on neonatal outcome multiple years after maternal exposure. Data on the degree of transfer of GLP-1 agonist and SGLT2 inhibitors to human milk could be obtained by means of a clinical study where this transfer is measured in the milk of mothers who stopped breastfeeding. Finally the degree in which the adverse effects of GLP-agonists on the neonate can be ascribed to maternal malnutrition could be assessed in multiple animal models where the female gets pregnant during either the catabolic stage of GLP-1 agonist exposure or after reaching a stable body weight.

## Strengths and limitations of this review

We have comprehensively reviewed the current body of evidence and have combined all data available to us regarding the safety of GLP-1 agonists and SGLT2 inhibitors during pregnancy and lactation. Our scope, in regards to the EMA/FDA data extracted by ourselves, was limited to Europe and the USA. Data from other continents could have provided us with a broader understanding of geographical differences, which may be related to varied genetic backgrounds.

We identified current gaps in knowledge. The major limitation of this review is the limited availability of both animal and human studies. The human studies only contained a small number of subjects and often lacked data on factors with a potential impact on neonatal outcomes such as BMI, age, smoking, drug treatment characteristics (dose and administration time) and trimester of exposure. The lack of follow-up of neonates born to women exposed to either GLP-1 agonists or SGLT2 inhibitors is a barrier to the study of potential adverse effects only apparent later in life.

We therefore suggest future studies to more thoroughly report maternal and treatment characteristics including trimester of exposure. Furthermore we suggest to update earlier studies by publishing follow-up data on neonatal outcome multiple years after maternal exposure.

## Conclusions

GLP-1 agonists reduce fetal survival, weight, growth and skeletal ossification and can induce major congenital anomalies. SGLT2 inhibitors cause dilation of the renal pelvis and tubules, congenital anomalies and an increased rate of miscarriages. Our findings broadly support the recommendation against use of these medications in both pregnancy and during lactation, in particular given effective and safe alternatives are available.

## Author contributions

DM: Screening and selection of studies, extraction of study data, contact with Lareb and the TIS of Switzerland, main author of the manuscript. DS: Proofreading/correcting manuscript assisting in drawing conclusions from the extracted data. AM: Creating the search synthax for Medline, Embase and Cochrane. FH: Proofreading/correcting manuscript assisting in drawing conclusions from the extracted data. RP: Proofreading/correcting manuscript assisting in drawing conclusions from the extracted data. SS: Screening and selection of studies, proofreading/correcting manuscript assisting in drawing conclusions from the extracted data. All authors contributed to the article and approved the submitted version.

## References

[1] Macintosh MCM Fleming KM Bailey JA Doyle P Modder J Acolet D. Perinatal mortality and congenital anomalies in babies of women with type 1 or type 2 diabetes in England, Wales, and Northern Ireland: population based study. BMJ (2006) 333(7560):177. doi: 10.1136/bmj.38856.692986.AE 16782722 PMC1513435

[2] Clausen TD Mathiesen E Ekbom P Hellmuth E Mandrup-Poulsen T Damm P. Poor pregnancy outcome in women with type 2 diabetes. Diabetes Care (2005) 28(2):323–8. doi: 10.2337/diacare.28.2.323 15677787

[3] Hadden DR Cull CA Croft DJ Holman RR. Poor pregnancy outcome for women with Type 2 diabetes. Diabetes Med (2003) 20(6):506–7. doi: 10.1046/j.1464-5491.2003.00955_2.x 12786690

[4] Cundy T Gamble G Townend K Henley PG MacPherson P Roberts AB. Perinatal mortality in Type 2 diabetes mellitus. Diabetes Med (2000) 17(1):33–9. doi: 10.1046/j.1464-5491.2000.00215.x 10691157

[5] de Valk HW van Nieuwaal NH Visser GH. Pregnancy outcome in type 2 diabetes mellitus: a retrospective analysis from the Netherlands. Rev Diabetes Stud (2006) 3(3):134–42. doi: 10.1900/RDS.2006.3.134 PMC1783588 17487337

[6] Hawthorne G Robson S Ryall EA Sen D Roberts SH Ward Platt MP. Prospective population based survey of outcome of pregnancy in diabetic women: results of the Northern Diabetic Pregnancy Audit, 1994. Bmj (1997) 315(7103):279–81. doi: 10.1136/bmj.315.7103.279 PMC2127206 9274546

[7] Yang J Cummings EA O'connell C Jangaard K. Fetal and neonatal outcomes of diabetic pregnancies. Obstet Gynecol (2006) 108(3 Pt 1):644–50. doi: 10.1097/01.AOG.0000231688.08263.47 16946226

[8] Zinman B Wanner C Lachin JM Fitchett D Bluhmki E Hantel S. Empagliflozin, cardiovascular outcomes, and mortality in type 2 diabetes. N Engl J Med (2015) 373(22):2117–28. doi: 10.1056/NEJMoa1504720 26378978

[9] Marso SP Daniels GH Brown-Frandsen K Kristensen P Mann JFE Nauck MA. Liraglutide and cardiovascular outcomes in type 2 diabetes. N Engl J Med (2016) 375(4):311–22. doi: 10.1056/NEJMoa1603827 PMC4985288 27295427

[10] Goldenberg RM Steen O. Semaglutide: review and place in therapy for adults with type 2 diabetes. Can J Diabetes (2019) 43(2):136–45. doi: 10.1016/j.jcjd.2018.05.008 30195966

[11] Shyangdan DS Royle P Clar C Sharma P Waugh N Snaith A. Glucagon-like peptide analogues for type 2 diabetes mellitus. Cochrane Database Syst Rev (2011) 2011(10):Cd006423. doi: 10.1002/14651858.CD006423.pub2 21975753 PMC6486297

[12] Ertugliflozin Drugs and Lactation Database (2023). Available at: https://www.ncbi.nlm.nih.gov/books/NBK500973/pdf/Bookshelf_NBK500973.pdf (Accessed 2023 20th of March).

[13] Hooijmans CR Rovers MM de Vries RBM Leenaars M Ritskes-Hoitinga M Langendam MW. SYRCLE’s risk of bias tool for animal studies. BMC Med Res Methodol (2014) 14:43. doi: 10.1186/1471-2288-14-43 24667063 PMC4230647

[14] Garcia-Flores V Romero R Miller D Xu Y Done B Veerapaneni C. Inflammation-induced adverse pregnancy and neonatal outcomes can be improved by the immunomodulatory peptide exendin-4. Front Immunol (2018) 9:1291. doi: 10.3389/fimmu.2018.01291 29967606 PMC6015905

[15] Graham DL Madkour HS Noble BL Schatschneider C Stanwood GD. Long-term functional alterations following prenatal GLP-1R activation. Neurotoxicol Teratol (2021) 87:106984. doi: 10.1016/j.ntt.2021.106984 33864929 PMC8555578

[16] Albiglutide EMA product information (2023). Available at: https://www.ema.europa.eu/en/documents/product-information/eperzan-epar-product-information_en.pdf (Accessed 2023 20th of March).

[17] Dulaglutide EMA product information (2023). Available at: https://www.ema.europa.eu/en/documents/product-information/trulicity-epar-product-information_en.pdf (Accessed 2023 20th of March).

[18] Exenatide EMA product information (2023). Available at: https://www.ema.europa.eu/en/documents/product-information/byetta-epar-product-information_en.pdf (Accessed 2023 20th of March).

[19] Liraglutide EMA product information (2023). Available at: https://www.ema.europa.eu/en/documents/product-information/victoza-epar-product-information_en.pdf (Accessed 2023 20th of March).

[20] Lixisenatide EMA product information. (2023). Available at: https://www.ema.europa.eu/en/documents/product-information/lyxumia-epar-product-information_en.pdf (Accessed 2023 20th of March).

[21] Semaglutide EMA product information. (2023). Available at: https://www.ema.europa.eu/en/documents/product-information/ozempic-epar-product-information_en.pdf (Accessed 2023 20th of March).

[22] Albiglutide FDA product information. (2023). Available at: https://www.accessdata.fda.gov/drugsatfda_docs/label/2017/125431s019lbl.pdf (Accessed 2023 20th of March).

[23] Dulaglutide FDA product information (2023). Available at: https://www.accessdata.fda.gov/drugsatfda_docs/label/2020/125469s036lbl.pdf (Accessed 2023 20th of March).

[24] Exenatide FDA product information (2023). Available at: https://www.accessdata.fda.gov/drugsatfda_docs/label/2009/021773s9s11s18s22s25lbl.pdf (Accessed 2023 20th of March).

[25] Liraglutide FDA product information (2023). Available at: https://www.accessdata.fda.gov/drugsatfda_docs/label/2017/022341s027lbl.pdf (Accessed 2023 20th of March).

[26] Lixisenatide FDA product information (2023). Available at: https://www.accessdata.fda.gov/drugsatfda_docs/label/2016/208471orig1s000lbl.pdf (Accessed 2023 20th of March).

[27] Semaglutide FDA product information (2023). Available at: https://www.accessdata.fda.gov/drugsatfda_docs/label/2017/209637lbl.pdf (Accessed 2023 20th of March).

[28] Talal Younes S Maeda KJ Sasser J Ryan MJ. The glucagon-like peptide 1 receptor agonist liraglutide attenuates placental ischemia-induced hypertension. Am J Physiol Heart Circ Physiol (2020) 318(1):H72–h77. doi: 10.1152/ajpheart.00486.2019 31729903 PMC6985807

[29] Diz-Chaves Y Toba L Fandiño J González-Matías LC Garcia-Segura LM Mallo F. The GLP-1 analog, liraglutide prevents the increase of proinflammatory mediators in the hippocampus of male rat pups submitted to maternal perinatal food restriction. J Neuroinflamm (2018) 15(1):337. doi: 10.1186/s12974-018-1370-7 PMC6282252 30518432

[30] Patti AM Giglio RV Pafili K Rizzo M Papanas N. Pharmacotherapy for gestational diabetes. Expert Opin Pharmacother (2018) 19(13):1407–14. doi: 10.1080/14656566.2018.1509955 30136869

[31] Greco D. Normal pregnancy outcome after first-trimester exposure to liraglutide in a woman with Type 2 diabetes. Diabetes Med (2015) 32(10):e29–30. doi: 10.1111/dme.12726 25683470

[32] Ivanišević M Herman M Horvatiček M Lovrenčić Vučić M Đelmiš J. Pregnancy outcome and liraglutide levels in serum and umbilical vein blood of a woman with type 2 diabetes. A case report. Gynaecol Perinatol (2018) 27(3-4):70–2.

[33] Williams J Pomeroy NE Pop-Busui R Lash R Douyon L Chames M. Case report: Exenatide use during pregnancy. Endocrinologist (2009) 19(3):119–21. doi: 10.1097/TEN.0b013e3181a5875e

[34] Hiles RA Bawdon RE Petrella EM. Ex vivo human placental transfer of the peptides pramlintide and exenatide (synthetic exendin-4). Hum Exp Toxicol (2003) 22(12):623–8. doi: 10.1191/0960327103ht402oa 14992323

[35] Canagliflozin EMA product information (2023). Available at: https://www.ema.europa.eu/en/documents/product-information/invokana-epar-product-information_en.pdf (Accessed 2023 20th of March).

[36] Dapagliflozin EMA product information (2023). Available at: https://www.ema.europa.eu/en/documents/product-information/forxiga-epar-product-information_en.pdf (Accessed 2023 20th of March).

[37] Empagliflozin EMA product information (2023). Available at: https://www.ema.europa.eu/en/documents/product-information/jardiance-epar-product-information_en.pdf (Accessed 2023 20th of March).

[38] Ertugliflozin EMA product information (2023). Available at: https://www.ema.europa.eu/en/documents/product-information/steglatro-epar-product-information_en.pdf (Accessed 2023 20th of March).

[39] Sotagliflozin EMA product information (2023). Available at: https://www.ema.europa.eu/en/documents/product-information/zynquista-epar-product-information_en.pdf (Accessed 2023 20th of March).

[40] Canagliflozin FDA product information (2023). Available at: https://www.accessdata.fda.gov/drugsatfda_docs/label/2017/204042s026lbl.pdf (Accessed 2023 20th of March).

[41] Dapagliflozin FDA product information (2023). Available at: https://www.accessdata.fda.gov/drugsatfda_docs/label/2021/202293s024lbl.pdf (Accessed 2023 20th of March).

[42] Empagliflozin FDA product information (2023). Available at: https://www.accessdata.fda.gov/drugsatfda_docs/label/2022/204629s033lbl.pdf (Accessed 2023 20th of March).

[43] Ertugliflozin FDA product information (2023). Available at: https://www.accessdata.fda.gov/drugsatfda_docs/label/2021/209803s004lbl.pdf (Accessed 2023 20th of March).

[44] Sotagliflozin FDA product information (2023). Available at: https://www.accessdata.fda.gov/drugsatfda_docs/label/2023/216203s000lbl.pdf (Accessed 2023 7th of September).

[45] Formoso G Ginestra F Di Dalmazi G Consoli A. Empagliflozin, metformin and insulin degludec, during pregnancy: a case report. Acta Diabetol (2018) 55(7):759–61. doi: 10.1007/s00592-018-1134-y 29594399

[46] Benhalima K Mathiesen ER Paldanius PM Mathieu C. The need for appropriate registration of pregnancy outcomes under newer oral glucose-lowering therapies. Diabetes Obes Metab (2018) 20(10):2477–80. doi: 10.1111/dom.13386 29806119

[47] Dulaglutide Drugs and Lactation Database (2023). Available at: https://www.ncbi.nlm.nih.gov/books/NBK500979/pdf/Bookshelf_NBK500979.pdf (Accessed 2023 20th of March).

[48] Exenatide Drugs and Lactation Database (2023). Available at: https://www.ncbi.nlm.nih.gov/books/NBK500978/pdf/Bookshelf_NBK500978.pdf (Accessed 2023 20th of March).

[49] Liraglutide Drugs and Lactation Database (2023). Available at: https://www.ncbi.nlm.nih.gov/books/NBK500977/pdf/Bookshelf_NBK500977.pdf (Accessed 2023 20th of March).

[50] Lixisenatide Drugs and Lactation Database (2023). Available at: https://www.ncbi.nlm.nih.gov/books/NBK500975/pdf/Bookshelf_NBK500975.pdf (Accessed 2023 20th of March).

[51] Semaglutide Drugs and Lactation Database (2023). Available at: https://www.ncbi.nlm.nih.gov/books/NBK500980/pdf/Bookshelf_NBK500980.pdf (Accessed 2023 20th of March).

[52] Wang J Johnson T Sahin L Tassinari MS Anderson PO Baker TE. Evaluation of the safety of drugs and biological products used during lactation: workshop summary. Clin Pharmacol Ther (2017) 101(6):736–44. doi: 10.1002/cpt.676 PMC5591026 28510297

[53] Canagliflozin Drugs and Lactation Database (2023). Available at: https://www.ncbi.nlm.nih.gov/books/NBK500623/pdf/Bookshelf_NBK500623.pdf (Accessed 2023 20th of March).

[54] Dapagliflozin Drugs and Lactation Database (2023). Available at: https://www.ncbi.nlm.nih.gov/books/NBK500971/pdf/Bookshelf_NBK500971.pdf (Accessed 2023 20th of March).

[55] Empagliflozin Drugs and Lactation Database (2023). Available at: https://www.ncbi.nlm.nih.gov/books/NBK500972/pdf/Bookshelf_NBK500972.pdf (Accessed 2023 20th of March).

[56] Brand KMG Saarelainen L Sonajalg J Boutmy E Foch C Vääräsmäki M. Metformin in pregnancy and risk of adverse long-term outcomes: a register-based cohort study. BMJ Open Diabetes Res Care (2022) 10(1). doi: 10.1136/bmjdrc-2021-002363 PMC8734020 34987051

[57] Lv S Wang J Xu Y. Safety of insulin analogs during pregnancy: a meta-analysis. Arch Gynecol Obstet (2015) 292(4):749–56. doi: 10.1007/s00404-015-3692-3 25855052

[58] Mank E Sáenz de Pipaón M Lapillonne A Carnielli VP Senterre T Shamir R. Efficacy and safety of enteral recombinant human insulin in preterm infants: A randomized clinical trial. JAMA Pediatr (2022) 176(5):452–60. doi: 10.1001/jamapediatrics.2022.0020 PMC8886453 35226099

[59] Glueck CJ Salehi M Sieve L Wang P. Growth, motor, and social development in breast- and formula-fed infants of metformin-treated women with polycystic ovary syndrome. J Pediatr (2006) 148(5):628–32. doi: 10.1016/j.jpeds.2006.01.011 16737874

[60] Sivan E Feldman B Dolitzki M Nevo N Dekel N Karasik A. Glyburide crosses the placenta in vivo in pregnant rats. Diabetologia (1995) 38(7):753–6. doi: 10.1007/s001250050348 7556974

[61] Schenker S Dawber NH Schmid R. Bilirubin metabolism in the fetus. J Clin Invest (1964) 43(1):32–9. doi: 10.1172/JCI104891 PMC289493 14105229

[62] Malek A Sager R Zakher A Schneider H. Transport of immunoglobulin G and its subclasses across the in vitro-perfused human placenta. Am J Obstet Gynecol (1995) 173(3 Pt 1):760–7. doi: 10.1016/0002-9378(95)90336-4 7573239

[63] Kandzija N Zhang W Motta-Mejia C Mhlomi V McGowan-Downey J James T. Placental extracellular vesicles express active dipeptidyl peptidase IV; levels are increased in gestational diabetes mellitus. J Extracell Vesicles (2019) 8(1):1617000. doi: 10.1080/20013078.2019.1617000 31164969 PMC6534242

[64] Griffiths SK. Placental structure, function and drug transfer. In: Campbell JP, editor. Continuing Education in Anaesthesia Critical Care & Pain Oxford University Press, vol. 15 (2015). p. 84–9.

[65] Fortunato SJ Menon RP Swan KF Menon R. Inflammatory cytokine (interleukins 1, 6 and 8 and tumor necrosis factor-alpha) release from cultured human fetal membranes in response to endotoxic lipopolysaccharide mirrors amniotic fluid concentrations. Am J Obstet Gynecol (1996) 174(6):1855–61. doi: 10.1016/S0002-9378(96)70221-1 8678151

[66] Lederman SA Rosso P. Effects of food restriction on fetal and placental growth and maternal body composition. Growth (1980) 44(2):77–88. 7439772

[67] Clark RL Robertson RT Peter CP Bland JA Nolan TE Oppenheimer L. Association between adverse maternal and embryo-fetal effects in norfloxacin-treated and food-deprived rabbits. Fundam Appl Toxicol (1986) 7(2):272–86. doi: 10.1016/0272-0590(86)90157-0 3758545

[68] Fleeman TL Cappon GD Chapin RE Hurtt ME. The effects of feed restriction during organogenesis on embryo-fetal development in the rat. Birth Defects Res B Dev Reprod Toxicol (2005) 74(5):442–9. doi: 10.1002/bdrb.20056 16193501

[69] Anderson GD Ahokas RA Lipshitz J Dilts PV Jr. Effect of maternal dietary restriction during pregnancy on maternal weight gain and fetal birth weight in the rat. J Nutr (1980) 110(5):883–90. doi: 10.1093/jn/110.5.883 7373434

[70] Blau JE Bauman V Conway EM Piaggi P Walter MF Wright EC. Canagliflozin triggers the FGF23/1,25-dihydroxyvitamin D/PTH axis in healthy volunteers in a randomized crossover study. JCI Insight (2018) 3(8). doi: 10.1172/jci.insight.99123 PMC5931122 29669938

[71] Tsugaya M Hayashi Y Sasaki S Kojima Y Kohri K Mogami T. Pseudo-Bartter syndrome without hypopotassemia: a case with unilateral multicystic dysplastic kidney and congenital contralateral hyronephrosis. Hinyokika Kiyo (1995) 41(1):51–5. 7900569

[72] Frazier KS. Species differences in renal development and associated developmental nephrotoxicity. Birth Defects Res (2017) 109(16):1243–56. doi: 10.1002/bdr2.1088 28766875

