# Supplementary material for: Effects of GLP-1 agonists and SGLT2 inhibitors during pregnancy and lactation on offspring outcomes: a systematic review of the evidence
Source: Front Endocrinol (Lausanne). 2023 Oct 10;14:1215356. doi: 10.3389/fendo.2023.1215356 (PMC10597691; doi:10.3389/fendo.2023.1215356)
Supplement: Supplementary file 1 [file Table_1.docx]

*Supplement 1. GLP-1 agonists, SGLT2 inhibitors and their availability.*

| **Name** | **Drug class** | **Available in Europe** | **Available in North America** |
| --- | --- | --- | --- |
| Exenatide | Exendin-4 based GLP-1 agonist | Yes | Yes |
| Lixisenatide | Exendin-4 based GLP-1 agonist | Yes | Yes |
| Albiglutide | Human GLP-1 based GLP-1 agonist | No | No |
| Dulaglutide | Human GLP-1 based GLP-1 agonist | Yes | Yes |
| Liraglutide | Human GLP-1 based GLP-1 agonist | Yes | Yes |
| Semaglutide | Human GLP-1 based GLP-1 agonist | Yes | Yes |
| Canagliflozin | SGLT2 inhibitor | Yes | Yes |
| Empagliflozin | SGLT2 inhibitor | Yes | Yes |
| Dapagliflozin | SGLT2 inhibitor | Yes | Yes |
| Ertugliflozin | SGLT2 inhibitor | Yes | Yes |
| Ipragliflozin | SGLT2 inhibitor | No | No |
| Luseogliflozin | SGLT2 inhibitor | No | No |
| Remogliflozin | SGLT2 inhibitor | No | No |
| Sergliflozin | SGLT2 inhibitor | No | No |
| Sotagliflozin | SGLT2 inhibitor | No | Yes* |
| Tofogliflozin | SGLT2 inhibitor | No | No |

** After manuscript submission, FDA approval was granted*

*Supplement 2. Key findings extracted from records on GLP-1 agonists and SGLT2 inhibitors on safety of drug exposure during pregnancy.*

| **Medication** | **Record** | **Species** | **Number exposed/ controls** | | **Timing/duration of exposure** | **Dose** | **Outcome assessment** |
| --- | --- | --- | --- | --- | --- | --- | --- |
| **GLP-1 agonists** |  |  |  | |  |  |  |
| Albiglutide | EMA  Product information^[32]^ | Mouse | Not described | | During organogenesis | 5 mg/kg/day | No adverse neonatal effects |
|  |  |  |  |  |  | 50 mg/kg/day | Embryo-fetal lethality, skeletal variations, reduced fetal weight, dehydration, coldness, and a delay in balanopreputial separation |
|  | FDA  Product information^[38]^ | Mouse | Not described | | Gestation day 6-15 (organogenesis) | 5 mg/kg/day | No adverse neonatal effects |
|  |  |  |  |  |  | 50 mg/kg/day | Embryo-fetal lethality, skeletal variations, reduced fetal weight, dehydration, coldness, and a delay in balanopreputial separation |
| Dulaglutide | EMA  Product information^[33]^ | Rat/rabbit | Not described | | Not described | Equivalent of 5-18 times the human clinical exposure of 4.5 mg/week | Skeletal effects, reduction in fetal growth, no fetal malformations. |
|  |  | Rat | Not described | | Throughout pregnancy and lactation | Equivalent of 7 times the human clinical exposure of 4.5 mg/week | Memory deficits in female offspring. |
|  | FDA  Product information^[39]^ | Rat | Not described | | During organogenesis | 2x MRHD* (0.49 mg/kg/3 days) | No adverse neonatal effects. |
|  |  |  |  |  |  | 6x MRHD* (1.63 mg/kg/3 days) | Reduced fetal weight associated with decreased maternal food intake and decreased weight gain. |
|  |  |  |  |  |  | 18x MRHD* (4.89 mg/kg/3 days) | Irregular skeletal ossifications and increases in post implantation loss. |
|  |  | Rabbit | Not described | | During organogenesis | 0.5x MRHD* (0.04 mg/kg/3 days) | No adverse neonatal effects. |
|  |  |  |  |  |  | 2x MRHD* (0.12 mg/kg/3 days) | No adverse neonatal effects. |
|  |  |  |  |  |  | 5x MRHD* (0.41 mg/kg/3 days) | Long lobular agenesis and skeletal malformations of the vertebrae and/or ribs in conjunction with decreased maternal food intake and decreased weight gain. |
| Exenatide | EMA  Product information^[34]^ | Mouse | Not described | | Mid-gestation | High doses (unspecified) | Skeletal effects and reduced fetal growth |
|  |  | Rabbit | Not described | | Mid-gestation | High doses (unspecified) | Reduced fetal growth |
|  | FDA  Product information^[40]^ | Mouse | Not described | | 2 weeks prior to and throughout mating until gestation day 7 | Systemic exposure up to 390x MRHD* | No adverse fetal effects |
|  |  | Mouse | Not described | | Gestation day 6-15 (organogenesis) | Systemic exposure 3x MRHD* and up | Reduced fetal and neonatal growth, cleft palate and skeletal effects. |
|  |  | Rabbit | Not described | | Gestation day 6-18 (organogenesis) | Systemic exposure 12x MRHD* and up | Irregular skeletal ossifications. |
| Exendin-4 | Garcia-Flores, V., et al. Front Immunol, 2018.^[13]^ | C57BL/6 (B6) Mouse | 4 groups of 3 dams injected intra-peritoneally with either lipopoly-saccharide (LPS), PBS, fluoresceine labelled Exendin-4 (FLEX), or LPS + FLEX.  Injection with the LPS component serves as an animal model of systemic inflammation. | | Gestation day 16 | 30 µg/kg injected intraperitoneally once | On gestation day 17 after mice were euthanized; exendin-4 was strongly detected in the uterus from mice injected with LPS and FLEX, and traces were detected in the decidua and fetal membranes of these injected mice. However exendin-4 was not detected in any of the fetal or maternal tissues of mice injected solely with FLEX. This indicates diffusion of Exendin-4 through the uterus and the maternal-fetal interface is only possible under systemic inflammation. |
| Exendin-4 | Graham, D.L., et al. Neurotoxicol Teratol, 2021.^[14]^ | C57Bl/6J Mouse | 4 pregnant dams exposed to exendin-4, 7 pregnant dams exposed to saline control. | | Daily injections 1 week before being bred up to parturition. | 10 µg/kg | No difference in litter size, maternal weight gain in pregnancy, no weight difference in pups at birth. A difference in weight is seen in the period after birth, where pups born to exendin-4 treated dams outweigh pups born to saline treated dams. This difference disappears after weaning. No fetal abnormalities. |
| Exenatide | Hiles, R.A., et al. Human & Experimental Toxicology, 2003.^[19]^ | Human | 3 placentas | |  | Mean concentration in the maternal compartment of 2009 ± 502 pg/mL | Fetal-to-maternal peptide concentration ratio of ≤ 0.017 during equilibrium perfusion of human placentas ex vivo. Therefore likely that maternal usage will result in negligible exposure to the fetus. |
| Exenatide | Williams, J., et al. Endocrinologist, 2009.^[18]^ | Human | 35 year old woman, with type 2 diabetes mellitus | | Multiple months prior to conception until the 14^th^ week of gestation. | Dosage unspecified | Fetal anatomical survey at 18 weeks: biometry consistent with dating. Amniotic fluid and visualized anatomy normal. No abnormalities on fetal echocardiography at 23 and 32 weeks. Normal fetal growth throughout pregnancy. E.Coli sepsis of infant diagnosed within hours of birth; 2835 g female, AS 1/1/2 successfully treated with intubation, resuscitation and antibiotic treatment. Normal weight and neurodevelopment at 2 month follow-up. |
| Liraglutide | EMA  Product information^[35]^ | Rat | Not described | | Mid-gestation | Highest dose (unspecified) | Slightly increased early embryonic death, reduced maternal weight and fetal/ neonatal growth with equivocal effect on ribs. |
|  |  | Rabbit | Not described | | Mid-gestation | Highest dose (unspecified) | Slightly increased early embryonic death, reduced maternal weight and fetal growth and skeletal variation. |
|  | FDA  Product information^[41]^ | Rat | Not described | | 2 weeks prior to mating through gestation day 17 | 0.8x MRHD* (0.1 mg/kg/day) | Fetal abnormalities and variations in kidney and blood vessels. Irregular ossifications of the skull and a more complete state of ossificiation.  Misshapen oropharynx and/or narrowed opening into larynx and umbilical hernia. |
|  |  |  |  |  |  | 3x MRHD* (0.25 mg/kg/day) | Fetal abnormalities and variations in kidney and blood vessels. Irregular ossifications of the skull and a more complete state of ossificiation. Umbilical hernia. |
|  |  |  |  |  |  | 11x MRHD* (1.0 mg/kg/day) | Fetal abnormalities and variations in kidney and blood vessels. Irregular ossifications of the skull and a more complete state of ossificiation. Slight increase in the number of early embryonic deaths, mottled liver and minimally kinked ribs. |
|  |  | Rabbit | Not described | | Gestation day 6-18  (organogenesis) | 0.01 mg/kg/day | Bilobed or bifurcated gallbladder, decreased fetal weight and major fetal abnormalities of the kidney and scapula. |
|  |  |  |  |  |  | ≥ 0.01 mg/kg/day | Bilobed or bifurcated gallbladder,  decreased fetal weight and major fetal abnormalities of the eyes and forelimb. |
|  |  |  |  |  |  | 0.025 mg/kg/day | Bilobed or bifurcated gallbladder,  decreased fetal weight and major fetal abnormalities of the brain, tail, sacral vertebrae, major blood vessels, heart and umbilicus. |
|  |  |  |  |  |  | ≥ 0.025 mg/kg/day | Bilobed or bifurcated gallbladder,  decreased fetal weight and major fetal abnormalities of the sternum. |
|  |  |  |  |  |  | 0.05 mg/kg/day | Bilobed or bifurcated gallbladder,  decreased fetal weight and major fetal abnormalities of the parietal bones and major blood vessels. |
| Liraglutide | Younes, S.T., et al. Am J Physiol Heart Circ Physiol, 2020.^[44]^ | CD-001 rat | 24 dams exposed to liraglutide, 21 exposed to saline control | | Gestation day 15-20 | 0.3 mg/kg/day | Significantly lowered pup weight and crown to rump length (as measured on gestation day 20). Correlation between reduced maternal intake and fetal demise. |
| Liraglutide | Greco, D. Diabet Med, 2015.^[16]^ | Human | 37 year old woman, with type 2 diabetes mellitus | | 2 years prior to conception until the 13^th^ week of gestation after which liraglutide was substituted with insulin. | 1.8 mg/day | Healthy female. Not macrosomic, normal APGAR score. Uneventful postpartum period except for transient neonatal hypoglycemia. No developmental abnormalities found after 3 months. |
| Liraglutide | Ivanisevic, M., et al. Gynaecologia et Perinatologia, 2018.^[17]^ | Human | 28 year old woman with type 2 diabetes mellitus; stabilized weight on liraglutide and 1 control (healthy pregnant woman not exposed to liraglutide) | | Multiple years prior to conception throughout the entirety of the pregnancy. | 1.8 mg/day | Healthy male. 4220 g, 51 cm long, AS 10/10. After delivery, 3.5 hour after Liraglutide was given, maternal and umbilical vein serum was extracted after which endogenous GLP-1 was removed thermally. Active GLP-1 ELISA found a liraglutide level of 8.4 pmol/L in maternal serum and 0.7 pmol/L in the umbilical vein blood which is below the sensitivity limit of the essay declared by the manufacturer of 2 pmol/L and was similar to the GLP-1 levels found in the umbilical vein blood of the control. |
| Liraglutide | Diz-Chaves, Y., et al. J Neuroinflammation, 2018.^[45]^ | Sprague-Dawley Rat | 12 dams exposed to liraglutide, 12 exposed to vehicle | | Gestational day 14 to parturition. | 100 µg/kg/12 h | No difference in body weight of pups at postnatal day 21. No difference in gestational length, litter size and sex ratio. No fetal abnormalities. |
| Lixisenatide | EMA  Product information^[36]^ | Rat | Not described | | Not described | 5x MRHD* | Malformations, growth retardation, ossification retardation and skeletal effects. Slight maternal toxicity consisting of low food consumption and reduced body weight. Reduced neonatal growth and slight increase in pup mortality. |
|  |  | Rabbit | Not described | | Not described | 32x MRHD* | Malformations, growth retardation, ossification retardation and skeletal effects. Slight maternal toxicity consisting of low food consumption and reduced body weight. |
|  | FDA  Product information^[42]^ | Rat | Not described | | Gestation day 6-17  (organogenesis) | 1x MRHD*  (5 mcg/kg/day) | Visceral closure defects and stunted growth. Impaired ossification associated with skeletal malformations. Decreases in maternal body weight, food consumption, and motor activity. |
|  |  |  |  |  |  | 70 mcg/kg/day | Visceral closure defects and stunted growth. Impaired ossification associated with skeletal malformations. Decreases in maternal body weight, food consumption, and motor activity. |
|  |  |  |  |  |  | 1000 mcg/kg/day | Visceral closure defects and stunted growth. Impaired ossification associated with skeletal malformations. Decreases in maternal body weight, food consumption, and motor activity. |
|  |  | Rabbit | Not described | | Gestation day 6-18  (organogenesis) | 6x MRHD*  (5 mcg/kg/day) | Multiple visceral and skeletal malformations, including closure defects. Decreases in maternal body weight, food consumption and motor activity. |
|  |  |  |  |  |  | 50 mcg/kg/day | Multiple visceral and skeletal malformations, including closure defects. Decreases in maternal body weight, food consumption and motor activity. |
|  |  |  |  |  |  | 500 mcg/kg/day | Multiple visceral and skeletal malformations, including closure defects. Decreases in maternal body weight, food consumption and motor activity. |
|  |  | Rabbit | Not described | | Organogenesis | 0.3 mcg/kg/day | No drug-related malformations |
|  |  |  |  |  |  | 2 mcg/kg/day | No drug-related malformations |
|  |  |  |  |  |  | 9x MRHD*  (5 mcg/kg/day) | No drug-related malformations |
| Semaglutide | EMA  Product information^[37]^ | Rat | Not described | | Not described | Not described | Embryotoxicity below clinically relevant exposures. Marked reductions in maternal body weight and reductions in embryonic survival and growth. Major skeletal and visceral malformations including effects on long bones, ribs, vertebrae, tail, blood vessels and brain ventricles. Impairment of the nutrient supply to the embryo across the rat yolk sac. |
|  |  | Rabbit | Not described | | Not described | Not described | Increased pregnancy loss and slightly increased incidence of fetal abnormalities. Maternal body weight loss of up to 16%. |
|  |  | Cynomolgus monkey | Not described | | Not described | Not described | Increased pregnancy loss and slightly increased incidence of fetal abnormalities. Maternal body weight loss of up to 16%. |
|  | FDA  Product information^[43]^ | Rat | Not described | | 2 weeks prior to mating through gestation day 17 in females.  4 weeks prior to mating in males. | 0.04x MRHD*  (0.01 mg/kg/day) | Reduced body weight gain and food consumption in parental animals. Reduced growth, visceral abnormalities of heart and blood vessels and skeletal abnormalities of cranial bones, vertebra and ribs in offspring (unclear at what dose these side effects emerge) |
|  |  |  |  |  |  | 0.1x MRHD*  (0.03 mg/kg/day) | Reduced body weight gain and food consumption in parental animals. Reduced growth, visceral abnormalities of heart and blood vessels and skeletal abnormalities of cranial bones, vertebra and ribs in offspring (unclear at what dose these side effects emerge) |
|  |  |  |  |  |  | 0.4x MRHD*  (0.09 mg/kg/day) | Reduced body weight gain and food consumption in parental animals. Reduced growth, visceral abnormalities of heart and blood vessels and skeletal abnormalities of cranial bones, vertebra and ribs in offspring (unclear at what dose these side effects emerge) |
|  |  | Rabbit | Not described | | Gestation day 6-19 | 0.01x MRHD*  (0.0010 mg/kg/day) | Reduced maternal body weight gain and food consumption. |
|  |  |  |  |  |  | 0.1x MRHD*  (0.0025 mg/kg/day) | Reduced maternal body weight gain and food consumption. Early pregnancy losses and minor visceral (kidney, liver) and skeletal (sternebra) fetal abnormalities. |
|  |  |  |  |  |  | 0.9x MRHD*  (0.0075 mg/kg/day) | Reduced maternal body weight gain and food consumption. Early pregnancy losses and minor visceral (kidney, liver) and skeletal (sternebra) fetal abnormalities. |
|  |  | Cynomolgus monkey | Not described | | Gestation day 16-50 | 0.4x MRHD*  (0.015 mg/kg twice weekly) | No drug related effects. |
|  |  |  |  |  |  | 2x MRHD*  (0.075 mg/kg twice weekly) | Maternal body weight loss and reduced body weight gain and food consumption, coinciding with sporadic abnormalities of vertebra, sternebra and ribs. |
|  |  |  |  |  |  | 6x MRHD*  (0.15 mg/kg twice weekly | Maternal body weight loss and reduced body weight gain and food consumption, coinciding with sporadic abnormalities of vertebra, sternebra and ribs. |
| **SLGT-2 inhibitors** | | | | | | | |
| Canagliflozin | EMA  Product information^[46]^ | Rat | Not described | | Not described | 19x clinical exposure dose | Ossification delays of metatarsal bones |
|  |  |  |  |  |  | 73x clinical exposure dose | Ossification delays of metatarsal bones |
|  | FDA  Product information^[51]^ | Rat | Not described | | 1st trimester of organogenesis | 100 mg/kg | No developmental toxicities |
|  |  |  |  |  | Postnatal day 21-90  (corresponding to 2nd and 3rd trimester of human renal development) | 4 mg/kg | Increased kidney weights and renal pelvic and tubular dilatation |
|  |  |  |  |  |  | 20 mg/kg | Increased kidney weights and renal pelvic and tubular dilatation |
|  |  |  |  |  |  | 65 mg/kg | Increased kidney weights and renal pelvic and tubular dilatation |
|  |  |  |  |  |  | 100 mg/kg | Increased kidney weights and renal pelvic and tubular dilatation |
|  |  | Rabbit | Not described | | 1st trimester of organogenesis | 160 mg/kg | No developmental toxicities |
| Canagliflozin | Benhalima, K., et al. Diabetes, Obesity and Metabolism, 2018.^[21]^ | Human | 29 Women; 14 with listed pregnancy outcomes | | Not described | Dosage unspecified | 1 congenital anomaly, 2 abortions, 1 fetal demise, 8 normal live births, 2 ongoing pregnancies. |
| Dapagliflozin | EMA  Product information^[47]^ | Rat | Not described | | Postnatal day 21-90  (corresponding to 2nd and 3rd trimester of human renal development) | ≥ 15x MRHD* | Increased incidence of renal pelvic and tubular dilatations in progeny. Increased kidney weights and macroscopic kidney enlargement. |
|  |  |  |  |  | Organogenesis | up to 1441x MRHD* | No maternal nor developmental toxicities |
|  |  | Rabbit | Not described | | Organogenesis | up to 1191x MRHD* | No maternal nor developmental toxicities |
|  | FDA  Product information^[52]^ | Rat | Not described | | Postnatal day 21-90  (corresponding to 2nd and 3rd trimester of human renal development) | 1 mg/kg/day | Increased kidney weights and increased incidence of renal pelvic and tubular dilatations |
|  |  |  |  |  |  | 15 mg/kg/day | Increased kidney weights and increased incidence of renal pelvic and tubular dilatations |
|  |  |  |  |  |  | 75 mg/kg/day | Increased kidney weights and increased incidence of renal pelvic and tubular dilatations |
|  |  |  | Not described | | Organogenesis | 75 mg/kg/day | No adverse effects |
|  |  |  |  |  |  | 150 mg/kg/ay | Maternal toxicities |
|  |  | Rabbit | Not described | | Organogenesis | 180 mg/kg/day | No adverse effects |
| Dapagliflozin | Benhalima, K., et al. Diabetes, Obesity and Metabolism, 2018.^[21]^ | Human | 21 Women; 8 with listed pregnancy outcomes | | Not described | Dosage unspecified | 1 congenital hydrocephalus, 2 encephalocele, 1 non-healthy infant with talipes, renal aplasia and oesophageal atresia, 2 induced abortions, 1 healthy premature infant, 1 healthy infant. |
| Empagliflozin | EMA  Product information^[48]^ | Rat | Not described | | Organogenesis | Unspecified maternally non-toxic doses | No adverse effects |
|  |  |  |  |  |  | Unspecified maternally toxic doses | Bent limb bones |
|  |  |  |  |  | pre- and postnatal studies | 1x MRHD* | No adverse effects |
|  |  |  |  |  |  | 4x MRHD* | Reduced weight gain of offspring |
|  |  | Rabbit | Not described | | Organogenesis | Unspecified maternally non-toxic doses | No adverse effects |
|  |  |  |  |  |  | Unspecified maternally toxic doses | Increased embryofetal loss |
|  | FDA  Product information^[53]^ | Rat | Not described | | Organogenesis | 48x MRHD*  (300 mg/kg/day) | No adverse developmental effects. |
|  |  |  |  |  |  | 154x MRHD*  (700 mg/kg/day) | Maternal toxicity. malformations of limb bones |
|  |  |  |  |  | Postnatal day 21-90  (corresponding to 2nd and 3rd trimester of human renal development) | 1 mg/kg/day | No adverse developmental effects. |
|  |  |  |  |  |  | 10 mg/kg/day | No adverse developmental effects. |
|  |  |  |  |  |  | 30 mg/kg/day | No adverse developmental effects. |
|  |  |  |  |  |  | 13x MRHD*  (100 mg/kg/day) | Increased kidney weights and renal tubular and pelvic dilatation. |
|  |  | Rabbit | Not described | | Organogenesis | 128x MRHD*  (300 mg/kg/day) | No adverse developmental effects. |
|  |  |  |  |  |  | 139x MRHD*  (700 mg/kg/day) | Maternal and fetal toxicity |
| Empagliflozin | Benhalima, K., et al. Diabetes, Obesity and Metabolism, 2018.^[21]^ | Human | 21 Women; 8 with listed pregnancy outcomes | | Not described | Dosage unspecified | 2 spontaneous abortions, 1 elective termination, 1 ectopic pregnancy, 3 healthy infants without congenital anomaly, 1 premature infant. |
| Empagliflozin | Formoso, G., et al. Acta Diabetol, 2018.^[20]^ | Human | 31 year old woman with type 2 diabetes mellitus | | Started an unspecified period before conception until the 5^th^ week of gestation. | 25 mg/day | Healthy female. 3280 g, 49 cm long, AS 8/9. Did not need intensive care unit support. No hypoglycemia, postnatal complications or congenital malformations were present. |
| Ertugliflozin | EMA  Product information^[49]^ | Rat | Not described | | Not described | 239x MRHD* | No adverse developmental effects |
|  |  |  |  |  |  | 510x MRHD* | Maternal toxicity, lower fetal viability and higher incidence of visceral malformation |
|  |  |  | Not described | | Postnatal day 21-90  (corresponding to 2nd and 3rd trimester of human renal development) | 13x MRHD* | Increased kidney weights, dilatation of the renal pelvis and tubules, and renal tubular mineralization. |
|  |  |  |  |  |  | 817x MRHD | Shorter femur length, increased trabecular bone in the femur and delayed puberty |
|  |  | Rabbit | Not described | | Not described | 1069x MRHD | No adverse developmental effects |
|  | FDA  Product information^[54]^ | Rat | Not described | | Postnatal day 21-90  (corresponding to 2nd and 3rd trimester of human renal development) | ≥ 5 mg/kg | Increased kidney weights, renal tubule and renal pelvis dilatation, and renal mineralization. |
|  |  |  | Not described | | Gestation day 6-17 | 50 mg/kg/day | No adverse developmental outcomes |
|  |  |  |  |  |  | 100 mg/kg/day | No adverse developmental outcomes |
|  |  |  |  |  |  | 707x MRHD  (250 mg/kg/day) | Reduced fetal viability, higher incidence of visceral malformation (membranous ventricular septal defect) |
|  |  | Rabbit | Not described | | Gestation day 7-19 | 50 mg/kg/day | No adverse developmental outcomes |
|  |  |  |  |  |  | 100 mg/kg/day | No adverse developmental outcomes |
|  |  |  |  |  |  | 250 mg/kg/day | Reduced fetal viability, higher incidence of visceral malformation (membranous ventricular septal defect) |
| Sotagliflozin | EMA  Product information^[50]^ | Rat | Not described | | Postnatal day 21-90  (corresponding to 2nd and 3rd trimester of human renal development) | 11x MRHD | Reversible renal tubular dilatation |
|  |  |  | Not described | | Not described | 350 mg/kg/day | Maternal toxicity |
|  |  |  |  |  |  | 158x MRHD  (400 mg/kg/day) | Embryo-lethality, fetal growth, cardiovascular and skeletal abnormalities. |
|  |  | Rabbit | Not described | | Not described | 9x MRHD  (200 mg/kg/day) | No adverse effects |
|  | FDA  Product information^[55]^ | Rat | | Not described | 1^st^ trimester of organogenesis in humans | 40x MRHD  (100 mg/kg/day) | No adverse effects |
|  |  |  |  |  |  | 161x MRHD  (350 mg/kg/day) | Embryo-lethality, effects on fetal growth, cardiovascular and skeletal abnormalities. |
|  |  | Rabbit | | Not described | 1^st^ trimester of organogenesis in humans | 9x MRHD  (200 mg/kg/day) | No adverse effects |

** MRHD: Maximum Recommended Human Dose.*

*Supplement 3. Key findings extracted from records on GLP-1 agonists and SGLT2 inhibitors on safety of drug exposure during lactation.*

| **Medication** | **Record** | **Species** | **Number exposed/ controls** | **Timing/duration of exposure** | **Dose** | **Outcome assessment** |
| --- | --- | --- | --- | --- | --- | --- |
| **GLP-1 agonists** |  |  |  |  |  |  |
| Albiglutide | EMA  Product information^[32]^ | Mouse | Not described | Not described | ≥1 mg/kg/day (equivalent to a below clinical human dose) | Reduced weight gain in pups while nursing. |
|  | FDA  Product information^[38]^ | Mouse | Not described | Lactation day 7-21 | ≥1 mg/kg/day (equivalent to a below clinical human dose) | Reduced weight gain in pups during the treatment period. |
| Dulaglutide | EMA  Product information^[33]^ | - | - | - | - | The Presence of dulaglutide in milk of treated of lactating animals was not described. |
|  | FDA  Product information^[39]^ | - | - | - | - | The presence of dulaglutide in milk of treated lactating animals was not determined. |
|  | Drugs and Lactation Database^[22]^ | Human  (theoretical) | - | - | - | Dulaglutide is a large peptide molecule; the amount in milk is likely to be very low. Probably destroyed in the infant’s gastrointestinal tract. |
| Exenatide | EMA  Product information^[34]^ | Mouse | Not described | Late gestation and lactation | High dose  (unspecified) | Reduced neonatal growth. |
|  | FDA  Product information^[40]^ | Mouse | Not described | Gestation day 6 through lactation day 20 | 3x MRHD*  (6 mcg/kg/day) | Increased number of neonatal deaths. Deaths were observed on postpartum days 2-4. Exenatide is present at low concentrations (≤ 2.5% concentration of maternal plasma following subcutaneous dosing). |
|  | Drugs and Lactation Database^[23]^ | Human  (theoretical) | - | - | - | Exenatide is a large peptide molecule; the amount in milk is likely to be very low. Probably destroyed in the infant’s gastrointestinal tract. |
| Liraglutide | EMA  Product information^[35]^ | Rat | Not described | Not described | Not described | Neonatal growth reduced while exposed to liraglutide. Unknown whether the reduced pup growth is caused by a reduced pup milk intake due to a direct GLP-1 effect or reduced maternal milk production due to decreased caloric intake. |
|  | FDA  Product information^[41]^ | Rat | Not described | Not described | Unspecified | Liraglutide was present unchanged in milk at concentrations of ± 50% of maternal plasma concentrations. |
|  | Drugs and Lactation Database^[24]^ | Human  (theoretical) | - | - | - | Liraglutide is a large peptide molecule; the amount in milk is likely to be very low. Probably destroyed in the infant’s gastrointestinal tract. |
| Lixisenatide | EMA  Product information^[36]^ | Rat | Not described | Late gestation and lactation | High dose  (unspecified) | Neonatal growth reduced in male rats exposed to liraglutide. Slight increase in pup mortality. |
|  | FDA  Product information^[42]^ | Rat | Not described | Not described | Unspecified | Low transfer (9.4%) of lixisenatide and its metabolites into milk and negligible (0.01%) levels of unchanged lixisenatide peptide in the gastric contents of weaning offspring. |
|  | Drugs and Lactation Database^[25]^ | Human  (theoretical) | - | - | - | Lixisenatide is a large peptide molecule; the amount in milk is likely to be very low. Probably destroyed in the infant’s gastrointestinal tract. |
| Semaglutide | EMA  Product information^[37]^ | Rat | Not described | Not described | Unspecified | Semaglutide was excreted in milk. |
|  | FDA  Product information^[43]^ | Rat | Not described | Not described | Unspecified | Semaglutide was detected in milk at levels 3-12 fold lower than in maternal plasma. |
|  | Drugs and Lactation Database^[26]^ | Human  (theoretical) | - | - | - | Semaglutide is a large peptide molecule; the amount in milk is likely to be very low. Probably destroyed in the infant’s gastrointestinal tract. |
| **SLGT-2 inhibitors** | | | | | | |
| Canagliflozin | EMA  Product information^[46]^ | Rat | Not described | Gestation day 6 - Postnatal day 20 | ≥ 5.9x MRHD*  (> 30 mg/kg/day) | Decreased body weights in male and female offspring. |
|  |  |  |  |  |  |  |
|  | FDA  Product information^[51]^ | Rat | Not described | Postpartum day 13 | Unspecified | Canagliflozin was present at a milk/plasma ratio of 1.40, indicating that it and its metabolites are transferred into milk at a concentration comparable to that in plasma. Juvenile rats directly exposed to canagliflozin showed increased risk of renal pelvic and tubular dilations. |
|  |  |  |  |  |  |  |
|  |  |  |  |  |  |  |
|  |  |  |  |  |  |  |
|  |  |  |  |  |  |  |
|  |  |  |  |  |  |  |
|  | Drugs and Lactation Database^[28]^ | Human  (theoretical) | - | - | - | Canagliflozin is uncharged and highly protein bound in plasma. Therefore unlikely to pass into breastmilk in clinically important amounts. |
| Dapagliflozin | EMA  Product information^[47]^ | Rat | Not described | Postnatal day 21 - postnatal day 90 | ≥ 15x MRHD* | Dose-related increases in kidney weight and macroscopic kidney enlargement. Renal pelvic and tubular dilatations, which were not fully reversible within the 1-month recovery period. |
|  |  |  |  |  |  |  |
|  |  |  |  |  |  |  |
|  | FDA  Product information^[52]^ | Rat | Not described | Not described | Unspecified | Dapagliflozin was present at a milk/plasma ratio of 0.49, indicating that it and its metabolites are transferred into milk at a concentration that is approximately 50% of that in maternal plasma. Juvenile rats directly exposed to dapagliflozin showed increased risk of renal pelvic and tubular dilatations. |
|  |  |  |  |  |  |  |
|  |  |  |  |  |  |  |
|  |  |  |  |  |  |  |
|  |  |  |  |  |  |  |
|  |  |  |  |  |  |  |
|  | Drugs and Lactation Database^[29]^ | Human  (theoretical) | - | - | - | Dapagliflozin is uncharged and highly protein bound in plasma. Therefore unlikely to pass into breastmilk in clinically important amounts. |
| Empagliflozin | EMA  Product information^[48]^ | Rat | Not described | Postnatal day 21 - postnatal day 60 | 11x MRHD*  (100 mg/kg/day) | Non-adverse, minimal to mild renal tubular and pelvic dilatation, reversible after 13-weeks recovery period. |
|  |  |  |  |  |  |  |
|  |  |  |  |  |  |  |
|  |  |  |  |  |  |  |
|  |  |  |  |  |  |  |
|  |  |  |  |  |  |  |
|  | FDA  Product information^[53]^ | Rat | Not described | Not described | Unspecified | The mean milk/plasma ratio ranged from 0.634 to 5. The mean maximal milk/plasma ratio of 5 occurred at 8 hours post-dose, suggesting accumulation of empagliflozin in the milk. Juvenile rats directly exposed to empagliflozin, showed increased risk of renal pelvic and tubular dilatations. |
|  |  |  |  |  |  |  |
|  |  |  |  |  |  |  |
|  |  |  |  |  |  |  |
|  |  |  |  |  |  |  |
|  |  |  |  |  |  |  |
|  |  |  |  |  |  |  |
|  |  |  |  |  |  |  |
|  | Drugs and Lactation Database^[30]^ | Human  (theoretical) | - | - | - | Empagliflozin is uncharged and highly protein bound in plasma. Therefore unlikely to pass into breastmilk in clinically important amounts. |
| Ertugliflozin | EMA  Product information^[49]^ | Rat | Not described | Postnatal day 21 - 90 | 13x MRHD* | Increased kidney weights, dilatation of renal pelvis and tubules and renal tubular mineralization. Not fully reversible after 1-month recovery period. |
|  |  |  |  |  | 817x MRHD* | Shorter femur length, increased trabecular bone in the femur. Not fully reversible after 1-month recovery period. |
|  | FDA  Product information^[54]^ | Rat | Not described | Postpartum day 10-12 | Unspecified | The mean milk/plasma ratio of ertugliflozin was 1.07. Juvenile rats directly exposed to ertugliflozin showed increased risk of increased kidney weight, renal mineralization and renal pelvic and tubular dilatations. |
|  |  |  |  |  |  |  |
|  |  |  |  |  |  |  |
|  |  |  |  |  |  |  |
|  |  |  |  |  |  |  |
|  |  |  |  |  |  |  |
|  |  |  |  |  |  |  |
|  | Drugs and Lactation Database^[31]^ | Human  (theoretical) | - | - | - | Ertugliflozin is uncharged and highly protein bound in plasma. Therefore unlikely to pass into breastmilk in clinically important amounts. |
| Sotagliflozin | EMA  Product information^[50]^ | Rat | Not described | Not described | 18x MRHD* for male offspring  31x MRHD* for female offspring | Available toxicological data have shown excretion of sotagliflozin in milk. Juvenile rats directly exposed to sotagliflozin showed no sotagliflozin-related toxicity. |
|  | FDA  Product information^[55]^ | Rat | Not described | Postnatal day 21-90 (corresponding to the late 2^nd^ and 3^rd^ trimester of human renal development) | 3 mg/kg/day | No adverse effects |
|  |  |  |  |  | 10 mg/kg/day | Dose-related increased kidney weight in males |
|  |  |  |  |  | 30 mg/kg/day | Dose-related increased kidney weight in males and females with renal tubular and pelvis dilation which was fully or partially reversed after a 29-day recovery period. |
|  |  |  |  |  | 75 mg/kg/day | Dose-related increased kidney weight in males and females with renal tubular and pelvis dilation which was fully or partially reversed after a 29-day recovery period. |

** MRHD: Maximum Recommended Human Dose.*

*Supplement 4. Search strategy Medline (Ovid)*

| # | Searches |
| --- | --- |
| 1 | exp Diabetes Mellitus/ |
| 2 | (diabet* or IDDM or T1DM or T1D or T2D or NIDDM).ti,ab,kf. |
| 3 | ((diabet* or dm) adj2 (type adj2 (one or "1" or I or two or "2" or II))).ti,ab,kf. |
| 4 | ((DM or diabet*) adj3 ("noninsulin dependent*" or "non insulin" or "adult onset" or "mature onset")).ti,ab,kf. |
| 5 | or/1-4 |
| 6 | exp Pregnancy/ or exp Maternal Health Services/ or exp Pregnant Women/ or exp Fetal Monitoring/ or exp Pregnancy Complications/ or exp Pregnancy Outcome/ or exp fetal death/ or exp fetal diseases/ or exp Breast Feeding/ or exp Lactation/ or exp Birth Weight/ or exp Premature Birth/ or exp Infant, Premature/ or exp Amniotic Fluid/ or exp Chorioamnionitis/ or exp embryonic structures/ or exp placenta/ or exp Placental Circulation/ |
| 7 | (parturition or ante natal or antenatal* or pre natal* or prenatal* or postnatal* or neonate* or "neo nate*" or newborn* or "new born*" or baby or babies or postpartum or "post partum" or "post natal*" or peripartum or "peri partum" or amnio* or "chorion* vill*" or cesarean or caesarean or cesarian or caesarian or cesarien or caesarien or newborn* or new born* or f?etal* or f?etus* or pregnan* or gestation or childbirth* or labo?r or "child bear*" or childbear* or gravidity or Eclampsia or preeclampsia or "HELLP Syndrome" or childbirth* or "child birth*").ti,ab,kf. |
| 8 | (pregnan* adj5 (wom?n or person* or complicat* or disorder* or outcome* or abnormal*)).ti,ab,kf. |
| 9 | ((premature* or "pre mature*" or preterm* or "pre term*") adj3 (infant* or pregnan* or labo?r or deliver* or "childbirth*" or "child birth*" or child* or baby or babies or neonate* or newborn* or "new born*" or abnormal*)).ti,ab,kf. |
| 10 | (breastfed or breastfeed or fore milk or hind milk or foremilk or hindmilk or mother* milk or lactat* or amniotic* or chorioamnionitis).ti,ab,kf. |
| 11 | (breast adj3 (feed* or milk or lactat*)).ti,ab,kf. |
| 12 | (birth* adj3 (weight* or lenght* or outcome* or complication* or disorder* or abnormal*)).ti,ab,kf. |
| 13 | (placent* or embryo* or zygote*).ti,ab,kf. |
| 14 | (eph adj3 (complex* or gestos* or toxemia*)).ti,ab,kf. |
| 15 | or/6-14 |
| 16 | exp Exenatide/ or exp Liraglutide/ |
| 17 | (Exenatide or Liraglutide or dulaglutide or albiglutide or lixisenatide or Semaglutide or bydureon or byetta or saxenda or victoza or albenatide or beinaglutide or "cjc 1131" or cotadutide or danuglipron or efpeglenatide or "exendin 4" or liraglutide or "ly 307161" or pegapamodutide or taspoglutide or tirzepatide).ti,ab,kf. |
| 18 | (("glp 1 receptor" or "glucagon like peptide 1 receptor" or "glp1 receptor") adj3 (agonist* or agent* or drug* or compound*)).ti,ab,kf. |
| 19 | or/16-18 |
| 20 | exp Canagliflozin/ or exp Sodium-Glucose Transporter 2 Inhibitors/ |
| 21 | (gliflozin or Canagliflozin or Invokana or dapagliflozin or Farxiga or Jardiance or empagliflozin or Steglatro or ertugliflozin or Ipragliflozin or Luseogliflozin or Luseogliflozin or Sergliflozin or Sergliflozin or Sergliflozin or Atigliflozin or bexaglifozin or "bi 44847" or enavogliflozin or ertugliflozin or licogliflozin or remogliflozin or sotagliflozin or tofogliflozin).ti,ab,kf. |
| 22 | (("sglt 2" or "sodium glucose transporter 2" or sglt2) adj3 (inhibitor* or agent* or drug* or compound*)).ti,ab,kf. |
| 23 | or/20-22 |
| 24 | 19 or 23 |
| 25 | 5 and 15 and 24 |

*Supplement 5. Search strategy Embase (Ovid)*

| # | Searches |
| --- | --- |
| 1 | exp diabetes mellitus/ |
| 2 | (diabet* or IDDM or T1DM or T1D or T2D or NIDDM).ti,ab,kf. |
| 3 | ((diabet* or dm) adj2 (type adj2 (one or "1" or I or two or "2" or II))).ti,ab,kf. |
| 4 | ((DM or diabet*) adj3 ("noninsulin dependent*" or "non insulin" or "adult onset" or "mature onset")).ti,ab,kf. |
| 5 | or/1-4 |
| 6 | exp pregnancy/ or exp pregnant woman/ or exp maternal health service/ or exp fetus monitoring/ or exp pregnancy disorder/ or exp "parameters concerning the fetus, newborn and pregnancy"/ or exp fetus death/ or exp fetus disease/ or exp breast feeding/ or exp lactation/ or exp drug exposure during lactation/ or exp prematurity/ or amnion fluid/ or placenta function/ or exp "functions of embryonic, fetal and placental structures"/ or exp "embryonic and placental structures"/ or exp embryo/ or exp placenta circulation/ |
| 7 | (parturition or "ante natal*" or antenatal* or "pre natal*" or prenatal* or postnatal* or postpartum or "post partum" or "post natal*" or peripartum or "peri partum" or amnio* or "chorion* vill*" or cesarean or caesarean or cesarian or caesarian or cesarien or caesarien or baby or babies or newborn* or "new born*" or neonate* or f?etal or f?etus or pregnan* or gestation or childbirth* or labo?r or "child bear*" or childbear* or gravidity or eclampsia or preeclampsia or "HELLP Syndrome" or childbirth* or "child birth*").ti,ab,kf. |
| 8 | (pregnan* adj5 (wom?n or person* or complicat* or disorder* or outcome* or abnormal*)).ti,ab,kf. |
| 9 | ((premature* or "pre mature*" or preterm* or "pre term*") adj3 (infant* or pregnan* or labo?r or deliver* or "childbirth*" or "child birth*" or child* or baby or babies or neonate* or newborn* or "new born*" or abnormal*)).ti,ab,kf. |
| 10 | (breastfed or breastfeed or fore milk or hind milk or foremilk or hindmilk or mother* milk or lactat* or amniotic* or chorioamnionitis).ti,ab,kf. |
| 11 | (breast adj3 (feed* or milk or lactat*)).ti,ab,kf. |
| 12 | (birth* adj3 (weight* or lenght* or outcome* or complication* or disorder* or abnormal*)).ti,ab,kf. |
| 13 | (placent* or embryo* or zygote*).ti,ab,kf. |
| 14 | (eph adj3 (complex* or gestos* or toxemia*)).ti,ab,kf. |
| 15 | or/6-14 |
| 16 | exp glucagon like peptide 1 receptor agonist/ |
| 17 | (Exenatide or Liraglutide or dulaglutide or albiglutide or lixisenatide or Semaglutide or bydureon or byetta or saxenda or victoza or albenatide or beinaglutide or "cjc 1131" or cotadutide or danuglipron or efpeglenatide or "exendin 4" or liraglutide or "ly 307161" or pegapamodutide or taspoglutide or tirzepatide).ti,ab,kf. |
| 18 | (("glp 1 receptor" or "glucagon like peptide 1 receptor" or "glp1 receptor") adj3 (agonist* or agent* or drug* or compound*)).ti,ab,kf. |
| 19 | or/16-18 |
| 20 | exp sodium glucose cotransporter 2 inhibitor/ |
| 21 | (gliflozin or Canagliflozin or Invokana or dapagliflozin or Farxiga or Jardiance or empagliflozin or Steglatro or ertugliflozin or Ipragliflozin or Luseogliflozin or Luseogliflozin or Sergliflozin or Sergliflozin or Sergliflozin or Atigliflozin or bexaglifozin or "bi 44847" or enavogliflozin or ertugliflozin or licogliflozin or remogliflozin or sotagliflozin or tofogliflozin).ti,ab,kf. |
| 22 | (("sglt 2" or "sodium glucose transporter 2" or sglt2) adj3 (inhibitor* or agent* or drug* or compound*)).ti,ab,kf. |
| 23 | or/20-22 |
| 24 | 19 or 23 |
| 25 | 5 and 15 and 24 |

*Supplement 6. Search* strategy Cochrane library

109 Trials matching "diabetes mellitus" or diabet* or IDDM or T1DM or T1D or T2D or NIDDM in Title Abstract Keyword AND pregnan* or fetus or fetal or newborn* or "breast feeding" or placent* or childbirth* or "child birth*"`or embryo or parturition or "ante natal*" or antenatal* or "pre natal*" or prenatal* or postnatal* or postpartum or "post partum" or "post natal*" or peripartum or "peri partum" or amnio* or "chorion* vill*" or cesarean or caesarean or cesarian or caesarian or cesarien or caesarien or baby or babies or newborn* or "new born*" or neonate* in Title Abstract Keyword AND "sodium glucose cotransporter 2 inhibitor" or "glucagon like peptide 1 receptor agonist" or Exenatide or Liraglutide or dulaglutide or albiglutide or lixisenatide or Semaglutide or bydureon or byetta or saxenda or victoza or albenatide or beinaglutide or "cjc 1131" or cotadutide or danuglipron or efpeglenatide or "exendin 4" or liraglutide or "ly 307161" or pegapamodutide or taspoglutide or tirzepatide or Exenatide or Liraglutide or dulaglutide or albiglutide or lixisenatide or Semaglutide or bydureon or byetta or saxenda or victoza or albenatide or beinaglutide or "cjc 1131" or cotadutide or danuglipron or efpeglenatide or "exendin 4" or liraglutide or "ly 307161" or pegapamodutide or taspoglutide or tirzepatide in Title Abstract Keyword - (Word variations have been searched)

*Supplement 7. SYRCLE’s risk of bias tool results*

| Record | Sequence generation | Baseline characteristics | Allocation concealment | Random housing | Blinded investigators | Random outcome assessment | Blinded result collection | Incomplete data justification | Unbiased conclusions | Other |
| --- | --- | --- | --- | --- | --- | --- | --- | --- | --- | --- |
| EMA product information; animal study data | Unclear | Unclear | Unclear | Unclear | Unclear | Unclear | Unclear | Unclear | Unclear | Unclear |
| FDA product information; animal study data | Unclear | Unclear | Unclear | Unclear | Unclear | Unclear | Unclear | Unclear | Unclear | Unclear |
| Garcia-Flores, V., et al. Front Immunol, 2018.^[13]^ | High | High | High | High | High | High | High | Low | Low | Low |
| Younes, S.T., et al. Am J Physiol Heart Circ Physiol, 2020.^[44]^ | Low | High | High | High | High | Low | High | Low | Low | Low |

*High: high risk of bias due to not satisfying requirements for limiting bias in domain, Low: low risk of bias due to satisfying requirements for limiting bias in domain, Unclear: bias unclear due to limited data on domain. The SYCLE tool does not recommend Individual summary scores as it is difficult to assign weights to specific domains.*
